# The ex vivo perfused mouse adrenal gland—a new model to study aldosterone secretion

**DOI:** 10.1007/s00424-024-02950-z

**Published:** 2024-03-28

**Authors:** Allein Plain, Laura Knödl, Ines Tegtmeier, Sascha Bandulik, Richard Warth

**Affiliations:** Medical Cell Biology, University of Regensburg, Regensburg, Germany

**Keywords:** Adrenal cortex, Mouse models, Aldosteronism, Secretion, Pathophysiology

## Abstract

Aldosterone is a steroid hormone that is important for maintaining the volume and ionic composition of extracellular fluids and is produced in the zona glomerulosa of the adrenal cortex. The basic mechanisms controlling aldosterone secretion are known. However, more detailed studies on the regulation of aldosterone secretion often fail due to the lack of suitable models: although secretion can be studied in cultured adrenocortical cells under defined conditions, the differentiation status of the cells is difficult to control and the complex anatomy of the adrenal cortex is lost. In living animals, the physiological context is intact, but the influences are manifold and the examination conditions cannot be sufficiently controlled. One method that closes the gap between cell models and studies in living animals is the isolated perfused adrenal gland. In the past, this method has provided important data on the pathophysiology of adrenal glands from larger animals, but the technique was not used in mice. Here, we developed a method for isolation and perfusion of the mouse adrenal gland to study aldosterone secretion. This technique preserves the complex anatomical and functional context of the mouse adrenal cortex, to ensure defined experimental conditions and to minimize extra-adrenal influences. Initial series of experiments with the ex vivo perfused mouse adrenal gland show that this model offers the possibility for unique insights into pathophysiological regulatory principles and is suitable for the use of genetically modified mouse models.

## Introduction

Via the secretion of steroid hormones, the adrenal cortex plays an important role in regulating various physiological processes in the body, including stress response, energy, and salt metabolism. The mineralocorticoid aldosterone is the focus of our interest. Its secretion is tailored to the body’s needs and must be precisely regulated [27]. Disturbances in this regulation are directly relevant to the development of diseases [13]. Aldosterone is synthesized and secreted in the outermost layer of the adrenal cortex, the zona glomerulosa. The secretion of aldosterone is stimulated by an increase in plasma K^+^ concentration or by angiotensin II when the renin–angiotensin–aldosterone system (RAAS) is activated. In the kidney, aldosterone induces the excretion of K^+^ and the reabsorption of Na^+^, making it important for the control of plasma electrolyte composition and blood pressure.

In aldosterone-producing adenomas (APA) of the adrenal cortex, the normal control of aldosterone secretion is impaired. Over the past 12 years, adrenal research has made important advances, and the discovery that adenomas and autonomous (regulation-independent) hormone secretion are associated with somatic mutations has significantly improved the understanding of adrenal pathophysiology [24, 35]. However, it is often difficult to elucidate in detail the consequences of a gene mutation, in particular the cytopathological changes, the underlying mechanisms, and the signaling pathways.

Physiologically, the regulation of aldosterone secretion by plasma K^+^ and angiotensin II is of central importance. However, important additional aspects of regulation are poorly understood, such as the adaptation of aldosterone secretion to osmolarity and salt intake or regulation by other hormones and mediators [5, 27].

A fundamental problem in researching the regulation of aldosterone secretion is the availability of suitable model systems: although cultured cells derived from adrenocortical carcinomas allow defined examination conditions, the degree of differentiation of the cells does not correspond to that of native glomerulosa cells and the environment of the cells does not correspond to the in vivo situation. On the other hand, the study of adrenal function in living animals is complicated by the poorly definable experimental conditions and the complexity of the living organism with its numerous compensatory mechanisms and secondary effects [3, 5–7, 14, 18, 29, 30].

Schneider and colleagues therefore developed an interesting model, the isolated perfused adrenal gland of the dog [23]. Using this model, they gained important insights into the regulation of aldosterone secretion, e.g., that angiotensin-stimulated aldosterone secretion is highly dependent on osmolarity and that even a small increase in osmolarity by 10 mosmol/l leads to a strong reduction in aldosterone secretion. Their results thus showed that the adrenal gland itself is an intrinsically osmo-sensitive organ and that osmolarity or plasma salinity strongly modulates aldosterone secretion independently of their effects on renin and angiotensin [19, 22, 23, 31, 32].

The aim of the current study was to develop a technique to isolate and perfuse the mouse adrenal gland. This technique allows to study the aldosterone secretion of glomerulosa cells ex vivo in a controlled and reproducible manner. Isolated from the animal’s central nervous system and from hormones and factors that control the adrenal gland, valuable insights into its intrinsic function and regulation can be gained. Researchers can also use this method to study the effects of drugs and the function of adrenal glands from transgenic mice.

## Material and methods

### Animals

C57BL6/J mice (3–6 month of age) were provided with food and water ad libitum and maintained on a 12-h light:12-h dark cycle. Before any manipulation or surgical procedure, mice were sacrificed by decapitation under deep anesthesia (2.5% isoflurane).

### Plastination of the blood vessels surrounding the right adrenal gland via vascular corrosion casting

To develop a suitable strategy for the perfusion and isolation of the right adrenal gland, it was necessary to determine the anatomical location of the arteries supplying the right adrenal gland with blood as accurately as possible in C57Bl6/J mice. Using a plastination technique (vascular corrosion molding [11]), we examined the anatomy of the right upper abdominal vessels including the blood supply to the right adrenal gland.

**Fig. 1:**
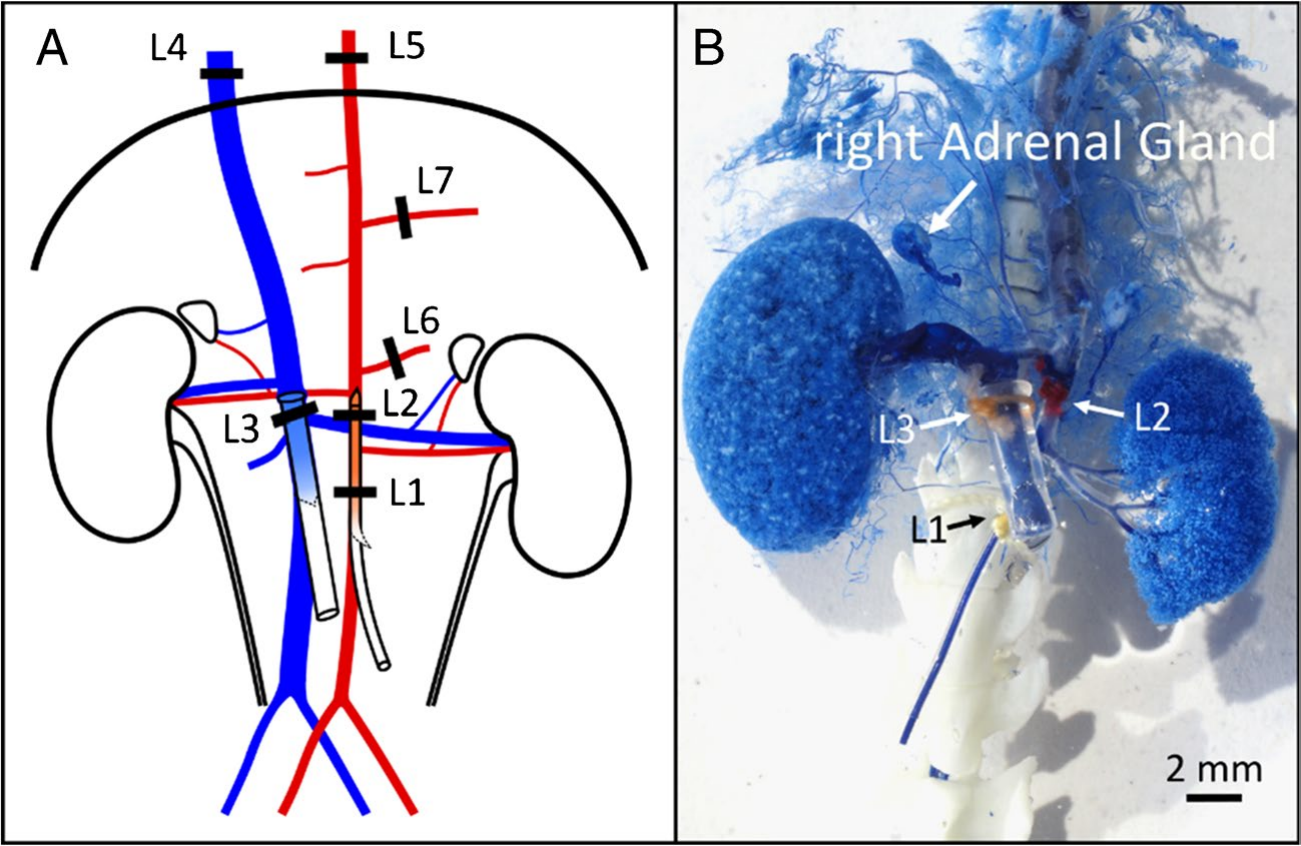
Vascular corrosion casting (plastination) of the right upper abdominal vessels. **A** Ligation scheme for plastination. L1 and L2 were placed strategically to hold the perfusing catheter in position. L3 was placed around the IVC to hold a collecting catheter, while L4 and L5 were placed respectively on the IVC or the aorta, cranial to the diaphragm to contain the resin. L6 and L7 blocked the flow to the mesenteric artery and the celiac trunk. **B** Picture of the blood vessels cast result after curation, corrosion, and extensive cleanup of all the capillary vessels that blocked the visibility of the arteries

The mice were anaesthetized with isoflurane and sacrificed by decapitation. The abdominal cavity and thoracic cavity were opened to gain access to the large arteries and veins. We followed the ligation scheme shown in Fig. 1 to preferentially perfuse the vessels on the right upper side of the abdominal cavity and prepare them for plastination. A perfusion catheter was inserted into the distal abdominal aorta through a transverse incision and fixed with ligatures 1 and 2. To later drain the epoxy resin from the tissue, another catheter was inserted into the inferior vena cava through a transverse incision and fixed with ligature 3.

The specimens were perfused with Ringer’s solution for a few minutes to flush out residual blood, then perfused with 3% PFA solution for 2–3 min and rinsed with Ringer’s solution for 5 min. During rinsing, 1 mL of BIODUR E20 Blue Resin (KEP05A1.0, BIODUR® Products GmbH) was mixed with 0.55 mL of BIODUR E20 Plus Hardener (KEH05A0.6, BIODUR® Products GmbH) to a final volume of 1.55 mL. The resin mixture was then perfused through the aortic catheter at a rate of approximately 1 mL/min. Once the blue resin began to flow from the catheter in the vena cava, perfusion of the resin was stopped and the preparation was allowed to rest for 1 h.

After 1 h of curing, the non-perfused soft tissues (i.e., intestine, stomach, pancreas, and spleen) and some parts of the liver were carefully removed. The specimen was then harvested and immersed in PBS for 24 h to complete the curing process. The organic matter adhering to the specimen was then digested and removed with 25% KOH for 3 to 5 days. The model was then rinsed with water, immersed in a water bath at 70 °C for 1 h, and then rinsed again. This procedure left only the blue resin, creating an anatomical image of the blood vessels in the perfused region of the abdominal cavity. A step-by-step protocol is shown in Table 1.

**Table 1:** Step-by-step protocol for the vascular corrosion casting (plastination) of the right upper abdominal vessels and adrenal blood supply

1	Sedate the mouse with isoflurane in a small dedicated gas chamber and euthanize by decapitation
2	Open the abdominal cavity with a ventral incision along the sagittal plane to minimize damage to the blood vessels
3	Place two ligatures (L1 and L2) around the aorta without closing them: one distal to the left renal artery (L1) and the other above the left renal vein and below the right renal artery (L2) (Fig. 1A)
4	Make a transversal incision in the aorta of around 2/3 of its diameter, distal to L1
5	Insert a pulled polyethylene catheter with heparinized Ringer’s solution flowing at around 1.5 mL/min
6	Move the catheter upwards until its opening reaches the right renal artery
7	Fix the catheter in place by closing the prepositioned ligature L1 around it
8	Reposition the tip of the aortic catheter, if necessary, to be just below the right renal artery, and tightly close ligature L2
9	Make a transversal incision in the inferior vena cava of 1/3 of its diameter, near the incision in the aorta
10	Place a third ligature (L3) around the inferior vena cava distal to the right renal vein but above the left renal vein, being extremely careful not to damage the aorta or the right renal artery. Do not close L3
11	Open the thorax in a V-shape: starting from the xiphoid cartilage. Separate the ventral segments of the ribs on both sides of the sternum up to near the shoulders
12	Lift the sternum to gain access to the thoracic cavity
13	Place a ligature (L4) around the vena cava, close to the cranial side of the diaphragm. Do not close L4 yet
14	Transect the esophagus (the aorta is below)
15	Place a ligature (L5) around aorta, on the cranial side of the diaphragm. Tightly close immediately
16	Place a ligature around the superior mesenteric artery (L6) and another around the celiac trunk (L7). Tightly close immediately
17	Insert into the inferior vena cava, via the incision previously made, a polyethylene catheter of 1 mm internal diameter and 2 mm external diameter, with a small flange at the opening and move it upwards to pass L3. The flange can be made by briefly presenting the end to fire
18	Firmly close L3 was around the catheter. The flange helps to avoid the catheter from sliding out
19	Close L4
20	Let the preparation be perfused with a Ringer’s solution for some more minutes to wash out any residual blood
21	Perfuse with 4% PFA for 2–3 min
22	Rinse with Ringer’s solution for 5 min
23	(While rinsing) Mix 10 parts of the BIODUR E20 blue resin with 5.5 parts of the BIODUR E20 Plus hardener to a final volume of 1.55 mL
24	Perfuse the resin mix via the aorta catheter using a 1-mL syringe at a rate of approximately 1 mL/min
25	Stop the perfusion once the blue resin began to flow out of the IVC catheter unmixed with Ringer’s solution
26	Allow to cure for 1 h with the syringe still attached to the aortic catheter (if removed, the negative pressure would compromise the quality of plastination)
27	After an hour of curing, carefully remove the soft tissues that were not perfused (i.e., intestines, stomach, pancreas, and spleen) as well as some parts of the liver
28	Cut out carefully the tissue block containing the area of interest
29	Immerse the tissue block in PBS for 24 h
30	Digest the preparation in 25% KOH for 3 to 5 days, with daily renewal of the 25% KOH
31	Rinse the KOH with double-distilled water
32	Bathe the preparation at 70 °C for 1 h, and then rinse again with double-distilled water. After this procedure, all remaining particles should have disappeared, leaving only an accurate cast of the blood vessels in the abdominal cavity. When performed correctly, the plastination process would go as deep as to cast very small capillary vessels
33	Use forceps and small scissors under a stereomicroscope to carefully and extensively remove as many as possible of the hepatic and muscular vessels and other capillaries that block the view of the arteries of interest

### Perfusion system

**Fig. 2:**
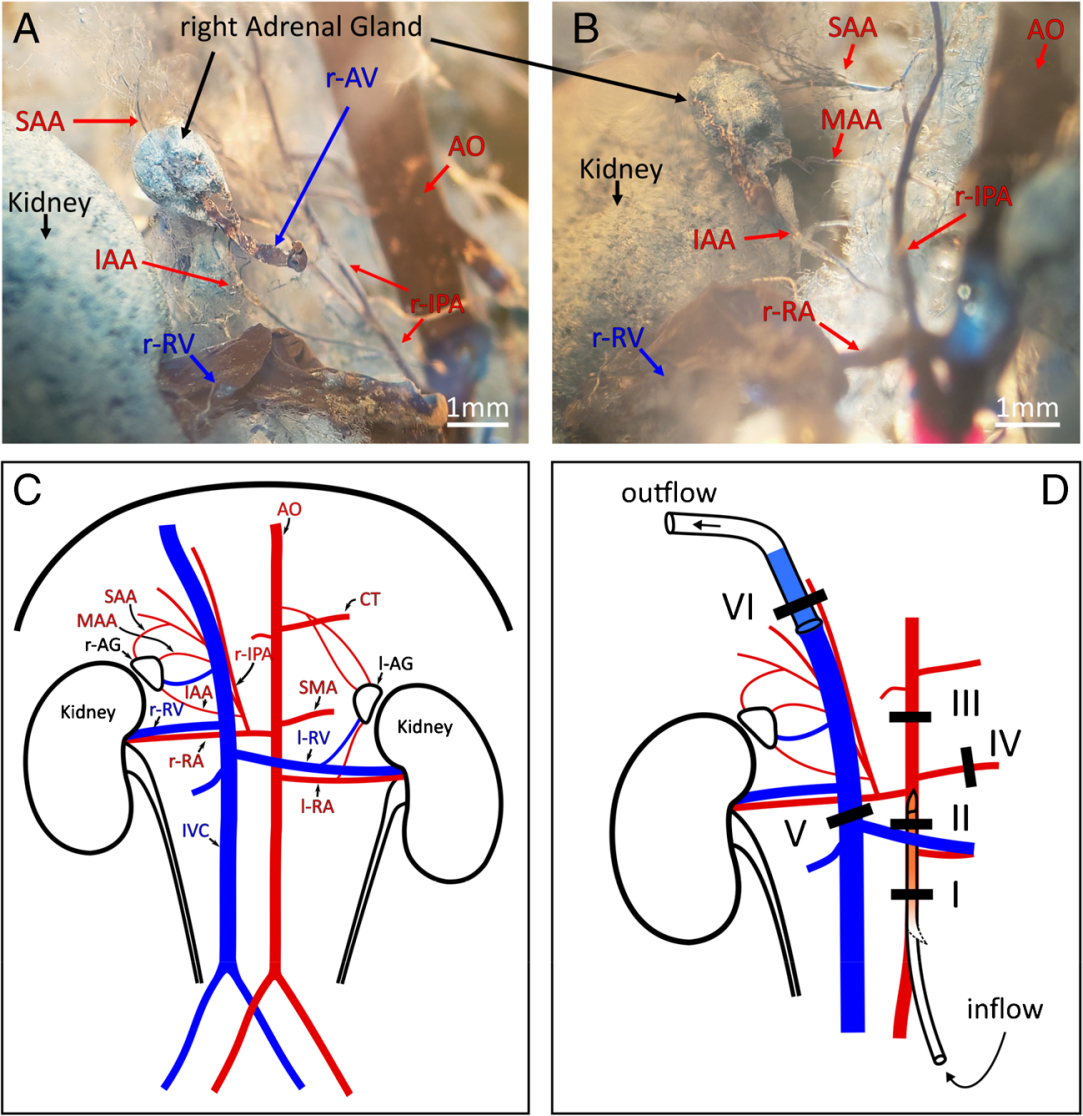
The right adrenal gland blood supply in the C57bl6/J mouse and ligation scheme for the perfusion.** A**, **B** Plastination of the blood vessels by vascular corrosion casting indicated that the three arteries that provided blood supply to the right adrenal gland originated from the right inferior phrenic artery. Panels **A** and **B** show different angles of the same cast, for better visibility. **C** Schematic representation of the blood vessels observed in the abdominal cavity with the performed casting procedure. **D** Ligation scheme to perfuse and isolate the right adrenal gland. Notice that ligature VI is simultaneously fixing the collecting catheter in place and squeezing the phrenic artery. SAA, superior adrenal artery; MAA, middle adrenal artery; IAA, inferior adrenal artery; AO, aorta; CT, celiac trunk; r-IPA, right inferior phrenic artery; r-AG and l-AG, right and left adrenal glands; SMA, superior mesenteric artery; r-RV and l-RV, right and left renal vein; r-RA and l-RA, right and left renal artery; IVC, inferior vena cava

**Fig. 3:**
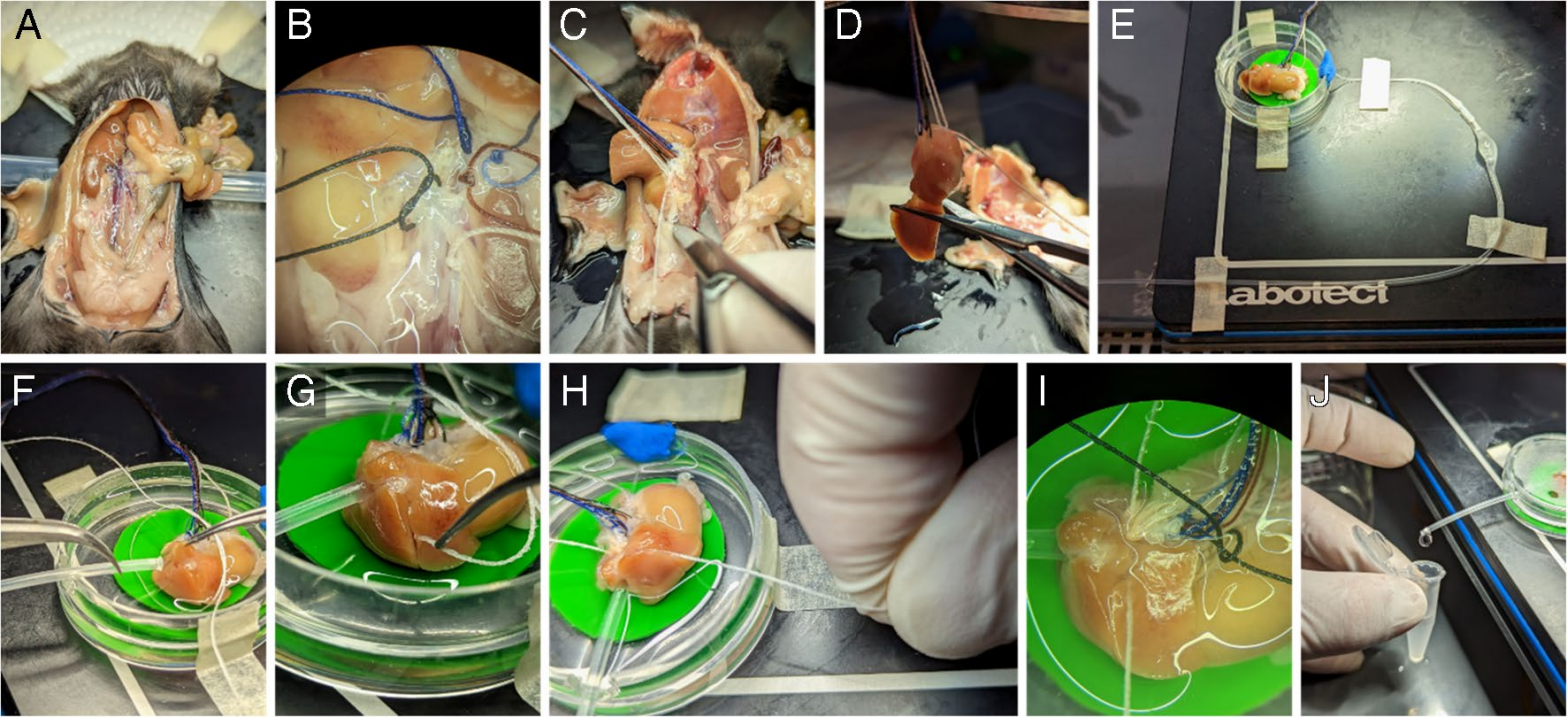
Key steps of the technique to isolate and perfuse the mouse right adrenal gland. **A** Abdominal cavity opened the to the mouse’s right side. **B** Ligatures I (white), II (red), III (blue), IV (light blue), and V (black) in position. Notice that ligature V was no yet closed. **C** About to start cutting out the right kidney/adrenal gland/liver while using the extra threads from the ligatures to gently guide the preparation out of the abdominal cavity. **D** Most of the left liver lobe was cut away, leaving behind the rest of the lobes. **E** The preparation transferred to a Petri dish adapted as incubation chamber. **F** Inserting the collecting catheter in the IVC via the liver, after having prepositioned ligature VI. **G** Ligature VI being placed between the right and the median liver lobes. **H** Tight closure of ligature VI around the liver, the muscle bundles of the diaphragmatic crura, and the collecting catheter. **I** The preparation after retracting the collecting catheter and shortly before closing ligature V. **J** Perfusate flowing out of the collecting catheter ready to be collected. Please note that the adrenal gland was covered by liver tissue and is not visible on these photos

We used a gravity-driven perfusion system with a pressure of 70 to 90 cm water column. The solution reservoirs were connected to the aortic catheter via a low-density polyethylene (LDPE) tube with an inner diameter of 1 mm and an outer diameter of 2 mm (339,653, Reichelt Chemietechnik). To produce the aortic catheter, the above-mentioned polyethylene tube was heated and stretched to an outer diameter of 0.5 to 1 mm. The aortic catheter was cut at a 45° angle to facilitate its insertion into the aorta. As a collecting catheter in the vena cava, we used a 3–4-cm-long tube segment, which was briefly flamed at one end to form a flange to allow fixation in the vessel by ligation (see Figs. 2 and 3). A bubble trap was placed in front of the perfusion catheter to minimize the risk of air bubbles entering the tissue.

### Surgical procedure

Based on the observations from the vascular corrosion cast, we developed a ligation scheme (Fig. 2D) to isolate and perfuse the right adrenal gland together with the right kidney. Mice were anesthetized with isoflurane and sacrificed by decapitation. The abdominal cavity was then opened (Fig. 3A), and two ligatures were placed around the aorta, ligature I and II (Fig. 2D). Ligations were performed using commercially available 100% cotton sutures with a diameter of approximately 300 µm. The abdominal aorta was then incised distal to ligature I and a polyethylene catheter filled with Ringer’s solution was inserted at the incision site. The catheter was pushed upward to pass through ligature II without crossing the outlet of the right renal artery. The ligatures were then tightened to hold the catheter in position. From then on, Ringer’s solution was continuously perfused through the catheter. An incision was made in the vena cava, distal to the site where ligature V would be placed, to facilitate the outflow of blood from the kidney and adrenal gland and reduce the risk of clot formation. A third and fourth ligature (Fig. 2D, ligatures III and IV, and Fig. 3B) were then placed and closed once they were in position: ligature III around the aorta, between the celiac trunk and the right renal artery, and ligature IV around the superior mesenteric artery, near the aorta. The thoracic cavity was then opened and the vena cava was cut near the diaphragm to remove the remaining resistance to perfusate outflow. Now, with the right kidney and adrenal gland perfused, ligature V was placed (but not closed) around the inferior vena cava distal to the right renal vein but cranial to the left renal vein (Figs. 2D and 3B), taking special care not to injure either the right renal artery or the aorta.

The specimen was then excised and placed in a small, custom-built chamber in which temperature (37 °C) and humidity were maintained (Fig. 3). To cut out the specimen, the mesenteric artery and the left renal vein were cut together with the left renal artery. The liver and gallbladder were then detached from the diaphragm. The protruding sutures of the ligatures were bundled and the tissue block consisting of the right kidney/adrenal gland/liver was carefully guided out of the abdominal cavity, whereby the musculature was cut as close as possible to the spine up to the diaphragm and finally the diaphragm was cut above the liver (Fig. 3C). After removal out of the animal, about 90% of the left lobe of the liver was removed (Fig. 3D) before the specimen was placed in the specially prepared incubation chamber (Fig. 3E). In the incubation chamber, the caudal liver lobe was localized and excised to expose the vena cava. The suture, which would be ligature VI, was then prepositioned below the liver and the collecting catheter was inserted into the inferior vena cava and carefully guided to the right renal vein (Fig. 3F). Ligature VI was then positioned between the right and middle lobes of the liver (Fig. 3G) and tightly closed with a surgeon’s knot around the liver, the right diaphragmatic muscle bundle, and the collecting catheter (Fig. 3H). The catheter was then withdrawn up to the ligature. To complete the procedure, ligature V was tightly closed (Fig. 3I).

The flow rate of the aortic catheter was adjusted to approximately 1.3 mL/min, which corresponds to the estimated physiological flow of the right renal artery, to ensure constant and physiological perfusion rate. If there were no obvious leaks, the kidney and adrenal gland were successfully perfused at this point, and the perfusate was collected via the catheter inserted into the inferior vena cava for further analysis (Fig. 3J). A step-by-step protocol can be found in Table 2; a video describes the procedure in detail (10.5283/epub.57910).

### Perfusion of adrenal glands and stimulation of aldosterone secretion

Isolated perfused adrenal glands were perfused with modified Ringer’s control solution (containing 128.76 mM NaCl, 2 mM KCl, 0.4 mM NaH_2_PO_4_, 1.6 mM Na_2_HPO_4_, 10 mM glucose, 1 mM MgSO_4_, 1.3 mM CaCl_2_, 5 mM HEPES, and 1% BSA; pH 7.4), constantly bubbled with oxygen. The stimulatory K^+^ concentrations were achieved by adding the proper amounts of an isotonic KCl stock solution (143.21 mM) containing 1.3 mM CaCl_2_ to not affect the calcium concentration. Experiments stimulating aldosterone secretion with angiotensin II (A9525, Sigma-Aldrich) were performed with Ringer’s control solution containing 4 mM KCI, and correspondingly 126.76 mM NaCl. Additionally, on those experiments with angiotensin II, 0.1 µM of forskolin (F6886, Sigma-Aldrich) was added to all solutions (including the control solution) in order to prevent malperfusion due to vasoconstriction. Aldosterone concentration was measured from 1 min collected perfusate using a specific ELISA (RE52301, IBL-International).

### Determination of tissue preservation and quality of perfusion

The adrenal glands were perfused with 2 mL Ringer’s control solution containing 60 µM propidium iodide (537059, Merck) and 0.25 µM Hoechst 33342 (62249, Thermo Fisher). After 5 min of incubation, the adrenal glands were rinsed with Ringer’s control solution for 2 to 3 min. The adrenal glands were then perfused with 3% PFA fixative solution for 2 min, harvested, and post-fixed in 3% PFA fixative solution for 30 min. They were then stored overnight in 1% PFA fixative solution containing 20% sucrose before being frozen and stored at − 80 °C. Subsequently, 10-µm-thick cryosections of these adrenal glands were prepared and examined with a Zeiss Axiovert inverted microscope or a Zeiss LSM 980 using the appropriate settings.

## Results

The aim of this study was to establish a method that allows the ex vivo perfusion of the mouse adrenal gland. Because of the vascular anatomy and accessibility, we chose the right adrenal gland for this purpose. As a basis for further planning of the surgical procedure, it was first necessary to examine the vascular supply of the right adrenal gland in C57BL6/J mice. For this purpose, we chose a plastination procedure in which the vessels supplying the adrenal gland were filled with a resin and the surrounding tissue could then be chemically removed to reveal the detailed vascular anatomy.

### Anatomy of blood supply to the mouse adrenal gland

The plastination specimens showed that the arterial blood supply of the right adrenal gland in C57Bl6/J mice originates in most cases from the right inferior phrenic artery. Three arteries branch off from the phrenic artery to the adrenal gland, the inferior, middle, and superior adrenal artery (Figs. 1 and 2A, B). A step-by-step protocol of the plastination is shown in Table 1. The anatomical information obtained has been summarized in the sketch shown in Fig. 2C. It is important to note that we observed variations in which the right inferior adrenal artery branches directly from the right renal artery, between the kidney and the right inferior phrenic artery; and the phrenic artery branches directly from the aorta rather than from the right renal artery in a very small percentage of mice. Similar observations have been made in humans [34]. This should be taken into account when attempting to isolate the right adrenal gland, as the right inferior phrenic artery should not be damaged.

### Challenges of the surgical procedure

Particular challenges during the surgical procedure were the complex arterial blood supply to the right adrenal gland (see above) and the aim of keeping the ischemia time as short as possible (3–5 min between the death of the animal and the perfusion of the oxygenated Ringer’s solution via the aortic catheter). Several ligatures were used to direct the flow so that only the adrenal gland and kidney were supplied with the oxygenated perfusate. Separating the kidney and adrenal gland was not practical due to their small size and the complex vascularization. However, as the kidney does not produce steroid hormones in any significant quantity and the blood supply to the kidney and adrenal gland is connected in parallel and not in series, additional perfusion of the kidney did not alter the amount of aldosterone secreted. Details of the surgical procedure can be found in the “Material and methods” section as well as in Figs. 1, 2, and 3, Table 2 and in a video describing the procedure in detail (10.5283/epub.57910).

**Table 2:** Step-by-step protocol for the isolation and perfusion of the right mouse adrenal gland

1	Sedate the mouse with isoflurane in a small dedicated gas chamber and euthanize via decapitation
2	Swiftly open the abdominal cavity to the mouse’s right side in a big D-shaped window
3	Place 2 ligatures in position around the aorta, ligatures I and II (figure). Ligature I, distal to the left renal artery, ligature II cranial to the left renal artery, distal to the right renal artery. (Make all ligatures, except for ligature VI, using square knots)
4	Make an incision in the aorta, distal to ligature I, and insert a catheter of similar diameter to the aorta with ringer solution flowing at around 1.3 mL/min
5	Move the catheter up inside the artery to pass ligature II, without surpassing the right renal artery, and close the ligatures to keep the catheter in place (the extra threads from each ligature will be used further in the procedure so, do not cut them out)
6	Make an incision on the cava distal to where ligature I is placed in the aorta
7	Place a third ligature (Fig. 2D, ligature III) around the aorta, distal to the celiac trunk, but cranial to the right renal artery. Close this ligature as soon as in position (the artery should be translucid and free from red blood cells at this point)
8	Place and close a fourth ligature (figure, ligature IV) around the superior mesenteric artery, in the vicinity of the aorta (at this point, the right kidney and the right adrenal gland are perfused, and the kidney should become pale)
9	Proceed by opening the chest cavity in V-shape from the xiphoid cartilage separating the ventral segments of ribs from both sides of the sternum. Cut the IVC near the diaphragm
10	Place, but do not close yet, ligature V around the IVC distal to the right renal vein but cranial to the left renal vein as illustrated; put special care not to damage the right renal artery nor the aorta
11	Cut out the preparation and transfer it to a small custom-made chamber where adequate temperature and moisture can be maintained • Cut the mesenteric artery and the left renal vein and artery, separating the left kidney and the intestines from our area of interest • Set apart the liver and gall bladder from the diaphragm • Hold all the extra threads from the ligatures in a bundle • Cautiously pull the threads bundle to help guide the preparation out of the abdominal cavity, and make an incision cutting the cava, aorta, and muscles distal to the aorta catheter • Carefully cut out the right kidney/adrenal gland/liver by cutting muscles as close to the spine as possible up to the diaphragm • Finally cut the diaphragm and the aorta above the liver • Cut and discard around 90% of the left liver lobe which will be hanging at the bottom, leaving behind the rest of the lobes • Transfer the preparation to the custom-made incubation chamber
12	Locate the rest of the left liver lobe and cut it out together with the caudal liver lobe, exposing the IVC
13	Preposition ligature VI around the liver and prepare a surgeon knot to later close the ligature. Let it wide open for now
14	Insert the collecting catheter, a PE tubing with a flange at the opening made by briefly presenting it to fire, into the IVC via the liver, and gently guide it up to close to the right renal vein
15	Close tightly ligature VI around the liver, the muscle bundles of the diaphragmatic crura, and the catheter, making sure that it runs between the right and the median lobes
16	Retract the collecting catheter until it the flange reaches the ligature (will stop it in position)
17	Close tightly ligature V, cut out the extra threads, and close the chamber
18	Start of the experimental protocol

### Controls to assess the quality of the perfusion and viability of the tissue

**Fig. 4:**
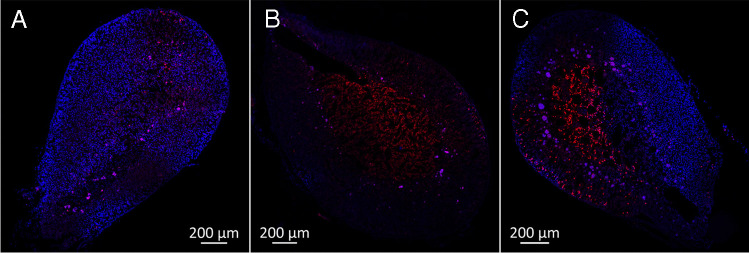
Control experiments to assess the quality of perfusion. Slices from isolated mouse adrenal glands that were perfused with Hoechst 33342 (blue) and propidium iodide (red) showcasing good (**A**), poor (**B**), and partial (**C**) perfusions

Propidium iodide and Hoechst 33342 were used to determine tissue preservation and perfusion quality. In contrast to Hoechst 33342, the DNA dye propidium iodide cannot reach the cell nucleus if the cell membrane is intact. Hoechst 33342 was used to determine the well-perfused tissue areas by staining the cell nuclei of all cells reached. In the perfused tissue areas, propidium iodide additionally stained the cell nuclei of cells that showed damage to the plasma membrane and thus acted as an indicator of dead cells in perfused areas. Sections from well-perfused adrenal glands appeared stained predominantly with Hoechst 33342 (Fig. 4A), covering most of the visible area. Conversely, slices from poorly perfused adrenal glands showed almost no staining, as neither dye reached the tissue (Fig. 4B). Sections from damaged adrenal glands or those perfused for too long showed increased propidium iodide staining. In some situations, partial perfusion was observed (Fig. 4C). As expected, we observed a certain correlation between the quality of perfusion and the responsiveness of the gland to stimuli of aldosterone secretion. However, this correlation was not always present: The extent of staining of the adrenal gland with Hoechst 33342 and propidium iodide at the end of an experiment, often lasting several hours, allowed only limited conclusions to be drawn about the perfusion quality at an earlier time point, as the perfusion quality may have decreased during the experiment, e.g., due to air embolism or progressive cell damage. On the other hand, adrenal glands that did not respond to stimulation at any time point were always poorly perfused. Overall, 70–80% of the adrenal glands responded to stimulation with K^+^ or angiotensin II at the end of the experiment, which was used as a measure of the success of the method.

### Ex vivo perfused adrenal gland: stimulation of aldosterone production with K^+^ and angiotensin II

**Fig. 5:**
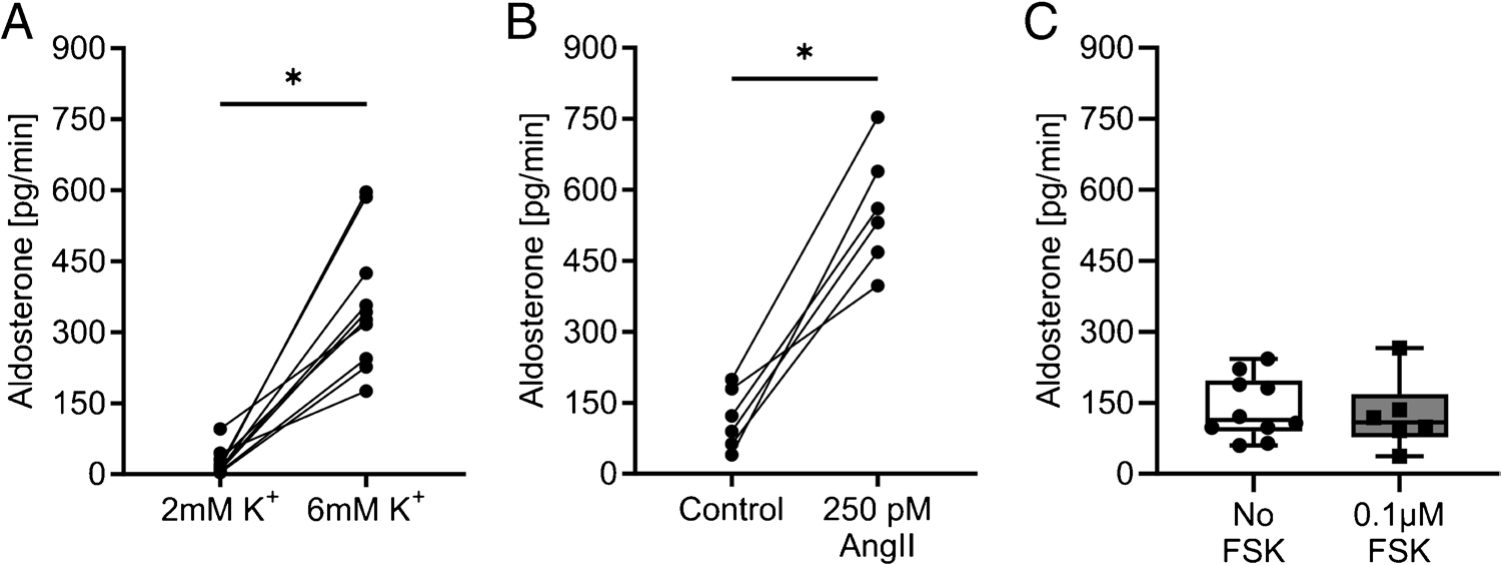
Stimulation of aldosterone secretion in isolated perfused mouse adrenal glands. **A** Aldosterone production in adrenal glands was stimulated with low (2 mM) and high K^+^ (6 mM) in the perfusate. **B** Aldosterone production in adrenal glands was stimulated with 250 pM angiotensin II (AngII). Control and AngII solutions contained 4 mM K^+^ and 0.1 µM forskolin. In **A** and **B**, the adrenal glands were perfused for 45 min with their respective control solution, followed by stimulation for 12 min. The stimulation sample was taken during 1 min at the end of the stimulation period. **C** Unpaired experiments showing the secretion of aldosterone in solutions containing 4 mM K^+^ with and without forskolin (FSK). Dots connected with lines were paired experiments; *p < 0.05

**Fig. 6:**
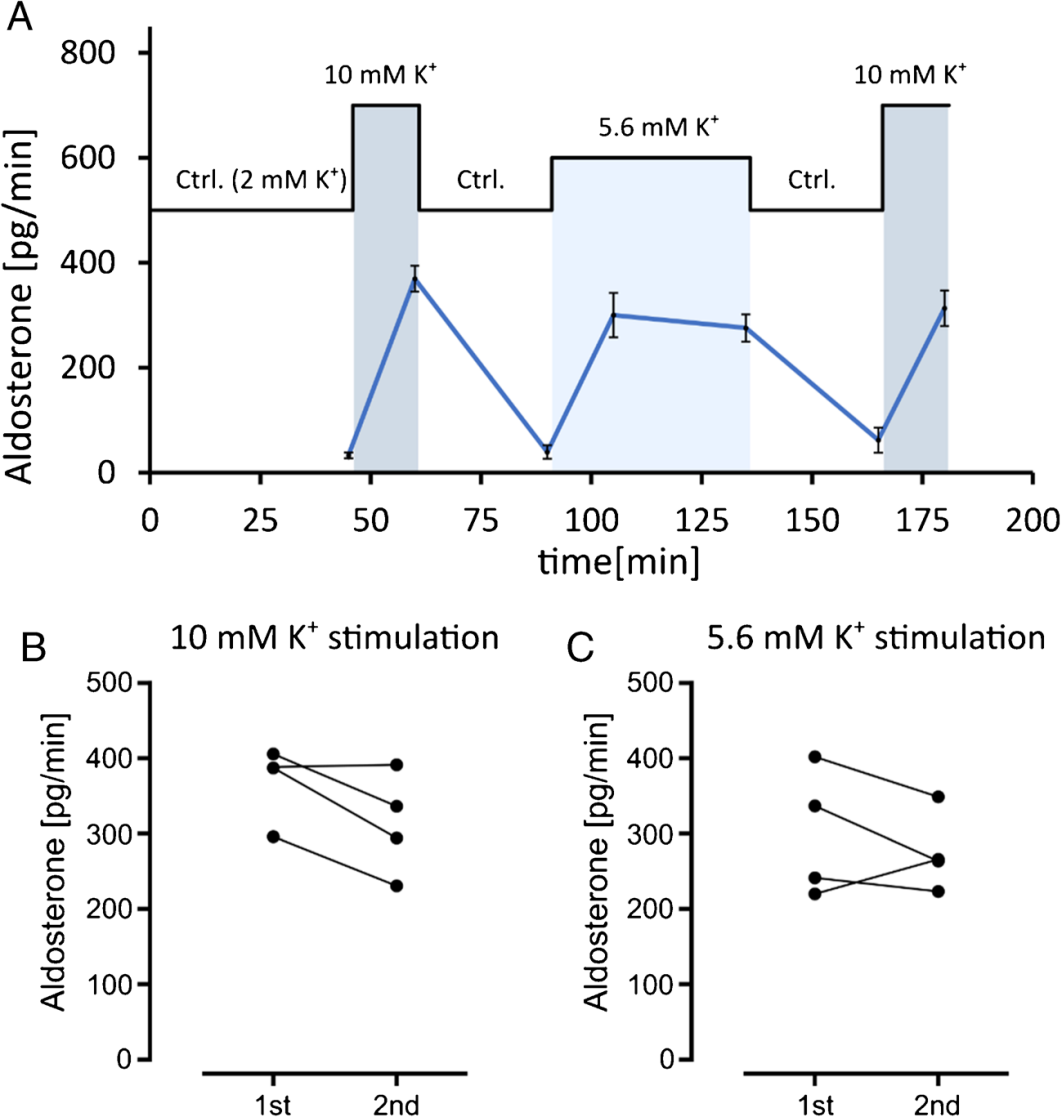
Effect of K^+^ on aldosterone secretion in the isolated perfused gland over a time course of 3 h. Aldosterone secretion in isolated perfused mouse right adrenal glands was sustained for at least 3 h after extraction of the organ with little to no loss of response to stimuli. **A** Time course and experimental protocol (mean values ± SEM, *n* = 4). **B** Data distribution and comparison between the first and second stimulations with 10 mM potassium solution as shown in **A**. **C** Data distribution and comparison between the first and second samples collected 30 min apart during stimulation with 5.6 mM K^+^ solution

Adrenal glands from C57B6/J mice were perfused in different series of experiments to investigate the responsiveness of zona glomerulosa cells to stimulation with K^+^ or angiotensin II (Figs. 5 and 6). The adrenal glands were first perfused with modified Ringer’s solution for 45 min before the perfusate was changed. This long control period was necessary to ensure a basal level of aldosterone secretion after possible stress of the animal before death and the stimulation of the tissue by mechanical alteration during the surgical procedure.

In the first two series of experiments, we investigated the effect of K^+^ and angiotensin II on aldosterone secretion by the isolated perfused adrenal glands. To investigate the stimulation with K^+^, the adrenal glands were perfused with a solution containing a low concentration of K^+^ (2 mM). Then, the adrenal glands were perfused with a solution containing a high (but still physiological) concentration of K^+^ (6 mM). Increasing the K^+^ concentration led to a pronounced stimulation of aldosterone secretion (Fig. 5A). For the investigation of the angiotensin II effect, solutions containing a physiological K^+^ concentration of 4 mM were used. We also added 0.1 µM forskolin to all solutions of the angiotensin series to mitigate angiotensin-induced vasoconstriction. Although the flow rate seems to influence mainly corticosterone secretion and less aldosterone secretion in the isolated perfused adrenal gland of the rat [16], we wanted to avoid possible ischemia-induced tissue damage and prevent angiotensin-mediated vasoconstriction by adding forskolin. In these experiments, stimulation with angiotensin II (250 pM) led to a significant increase in aldosterone secretion (Fig. 5B). Forskolin at the concentration of 0.1 µM appeared to have no relevant effect on aldosterone secretion (Fig. 5C).

In the next series of experiments, we investigated the reproducibility of the K^+^ stimulation and secretion of aldosterone over time to test how long experiments can be if valid results are to be obtained. It was remarkable that the perfused adrenal glands showed consistent and reversible responses to stimulation with high K^+^ concentrations over a time course of at least 3 h (Fig. 6).

## Discussion

The aim of this study was to develop a method for ex vivo perfusion of the mouse adrenal gland that provides unique insights into the pathophysiology and regulation of aldosterone secretion. The method of the isolated perfused adrenal gland has already been established for some animal models, e.g., retrograde [8] and orthograde [26] perfusion of the rat adrenal gland and the isolated perfused adrenal gland of the cat [10, 17] and the dog [23]. These studies have provided important data on the regulation of hormone secretion from the adrenal medulla and adrenal cortex. The regulation of aldosterone secretion, which is the focus of our interest, was intensively investigated in the isolated perfused adrenal gland of the dog [23]. Schneider and his colleagues used this model to gain exciting insights into the physiology of aldosterone secretion and the interaction of stimulation pathways [19, 33]. Among other things, they discovered that the adrenal gland of the dog has an intrinsic osmosensitivity [19, 21–23, 31, 32]. A number of transgenic mouse models have been developed to study the development and pathophysiology of the adrenal gland [1, 2, 4, 6, 12, 15]. The phenotype of these mice is usually studied at the animal level using in vivo methods, clinical chemistry methods, histologic techniques, fresh adrenal gland slices, and primary cultures. However, it is difficult to detect altered regulation of aldosterone secretion with these techniques, as too many parameters are involved in the living animal and the physiological context is no longer sufficiently given in isolated cells and tissues. To our knowledge, however, there is still no method for perfusing the mouse adrenal gland ex vivo.

The method of the isolated perfused mouse adrenal gland presented here is an adaptation of the method used for the perfusion of the mouse kidney [9, 25]. A particular challenge was the small size of the mouse adrenal gland and of the arteries that supply the mouse adrenal gland with blood. In the following, we will discuss the limitations and advantages of this technique:

### Challenges and limitations

- The preparation is time-consuming and requires a trained and experienced experimenter. The success rate depends on methodological expertise. The time that elapses between the killing of the animal and the perfusion of oxygenated Ringer’s solution via the aortic catheter is particularly critical. With sufficient training, this ischemia time is in the range of 3–5 min. Once aortic perfusion has been established and the fluid has been drained via the vena cava, it is the visualization of the vessels and the placement of the ligatures that require practice. The formation of thrombi and air embolisms must be avoided. A video describing the procedure in detail can be found here: 10.5283/epub.57910.
- Ischemia and technical problems can lead to impaired aldosterone secretion and thus to misinterpretations. We used a secretory stimulus at the end of the experiment as an indicator of successful perfusion and preserved tissue viability. Based on this evidence, our success rate was in the range of 70–80%. This success rate is acceptable for numerous applications. However, it is necessary to identify and exclude the experiments that were not successful. Otherwise, the scatter of the data and the risk of misinterpretation increase considerably and the number of experiments must be increased.
- Due to the small size of the mouse adrenal gland and its blood vessels, we have revised the original plan to remove the adrenal gland without other organs. Instead, we removed the right adrenal gland en bloc with the right kidney and part of the liver. The liver and kidney do not produce aldosterone and the blood supply to the adrenal gland is not downstream of the blood supply to the liver and kidney, but parallel to it. We therefore assume that the aldosterone secretion rate measured in the collecting catheter in the vena cava is not affected by the parallel blood supply to the kidney. For research questions other than aldosterone or corticosterone secretion, the collection of a mixture of fluids that have passed through the adrenal gland and kidney could be a problem.
- High concentrations of K^+^ and angiotensin II trigger vasoconstriction of the small arteries that supply the adrenal gland with blood, which can lead to ischemia in the downstream tissue. This should be taken into account when planning the experiments. We used forskolin to prevent vasoconstriction. At the concentration of 0.1 µM, forskolin appeared to have no relevant effect on aldosterone secretion (Fig. 5C). However, it should be borne in mind that vasodilating agents can influence aldosterone secretion, e.g., nitric oxide [20].

### Advantages

- Compared to cell culture, the degree of differentiation of the cells, the tissue architecture, and the cellular context are preserved. This is probably also the reason why the signal-to-noise ratio is very favorable, the measurements are very sensitive, and even small effects on the absolute amount of aldosterone secreted can be made visible. The classical stimuli of aldosterone secretion, K^+^ and angiotensin, are effective in the physiological concentration range. In cultured cells, however, non-physiologically high concentrations are usually necessary to induce comparable aldosterone secretion.
- The composition of the perfusate can be precisely defined. Due to the good definability of the experimental conditions, isolated perfused adrenal glands are very well suited for investigating transcriptional changes in response to a stimulus or for testing the effect of drugs on aldosterone secretion.
- The method is ideal for investigating rapid effects on aldosterone secretion (Fig. 6). Studies on the isolated perfused adrenal gland of the dog and on living mice have already shown that aldosterone secretion increases very rapidly after a stimulus [23, 28]. This increase in aldosterone secretion is so rapid (after 10 min) that it can hardly be explained by transcription and translation of Cyp11b2. The focus of the literature on the transcription of Cyp11b2 as a key factor in the regulation aldosterone secretion is justified when longer-term stimulation is considered (as typically used in cell culture). Physiologically, however, the rapid response to stimuli is also likely to be of considerable relevance and can be studied in more detail using the method of the ex vivo perfused mouse adrenal gland.
- Compared to living animals, a large number of influences and compensation pathways are eliminated. Influence by the vegetative nervous system is excluded. It is therefore possible to investigate which regulation is actually an intrinsic property of the adrenal gland and which mediators are directly effective in the adrenal gland. Moreover, it is possible to study the adrenal glands of genetically modified animals without having to consider secondary effects from other organ systems. Long experiments (at least over 3 h) with many data points are possible and thus paired experiments that allow good comparability of effects. This allows unique insights into the complex regulation and the time course of aldosterone secretion.
- By using animals that have already been killed, the isolated perfused adrenal gland does not involve manipulation of the living animal. This reduces ethical concerns and facilitates approval procedures.

## Conclusion

We have developed a technique for the perfusion of the adrenal glands of mice that has a good success rate after sufficient training and offers the possibility to directly examine the responses of the adrenal glands to stimuli. It provides a physiological method to study aldosterone secretion in the adrenal gland without the confounding effects and compensations that occur in living animals. The ex vivo method, unlike the in vivo method, is not a live animal experiment and is accessible to researchers who wish to apply it to genetically modified mouse models. The method also closes a previously existing gap between experiments on cultured adrenal cells and in vivo studies and enables a more comprehensive assessment of the pathophysiology of the adrenal cortex. Thus, this method offers the opportunity to gain unique insights into the intrinsic sensitivity of mouse adrenal glands and the (short-term) regulation of aldosterone secretion.

## Data Availability

Data is provided within the manuscript. A video describing the procedure in detail can be found here: 10.5283/epub.57910.
